# Enhanced subliminal emotional responses to dynamic facial expressions

**DOI:** 10.3389/fpsyg.2014.00994

**Published:** 2014-09-09

**Authors:** Wataru Sato, Yasutaka Kubota, Motomi Toichi

**Affiliations:** ^1^The Hakubi Project, Primate Research Institute, Kyoto UniversityInuyama, Japan; ^2^The Organization for Promoting Research in Developmental DisordersKyoto, Japan; ^3^Health and Medical Services Center, Shiga UniversityHikone, Japan; ^4^Faculty of Human Health Science, Graduate School of Medicine, Kyoto UniversityKyoto, Japan

**Keywords:** affective priming, emotional facial expressions, dynamic presentations, subliminal stimulation, unconscious emotion

## Abstract

Emotional processing without conscious awareness plays an important role in human social interaction. Several behavioral studies reported that subliminal presentation of photographs of emotional facial expressions induces unconscious emotional processing. However, it was difficult to elicit strong and robust effects using this method. We hypothesized that dynamic presentations of facial expressions would enhance subliminal emotional effects and tested this hypothesis with two experiments. Fearful or happy facial expressions were presented dynamically or statically in either the left or the right visual field for 20 (Experiment 1) and 30 (Experiment 2) ms. Nonsense target ideographs were then presented, and participants reported their preference for them. The results consistently showed that dynamic presentations of emotional facial expressions induced more evident emotional biases toward subsequent targets than did static ones. These results indicate that dynamic presentations of emotional facial expressions induce more evident unconscious emotional processing.

## INTRODUCTION

The induction of emotion in the absence of conscious awareness of the stimulus evoking such emotion has long been a subject of great interest (Pribram and Gill, 1976; Zajonc, 1980). Because such emotional processing may circumvent the supervision of consciousness, it may have great impact on our daily social behaviors (Ferguson and Bargh, 2004). From an evolutionary perspective, emotional processing without conscious awareness compared with processing with conscious awareness would be expected to have longer history and to confer an adaptive advantage by facilitating rapid and immediate reactions to biologically significant stimuli, such as predators.

Several behavioral studies have used subliminal presentations of emotional facial expressions in the context of the affective priming paradigm to investigate unconscious emotional processing (e.g., Murphy and Zajonc, 1993; Murphy et al., 1995; Rotteveel et al., 2001; for reviews, see Eastwood and Smilek, 2005; Winkielman, 2010). In a typical use of this paradigm, a facial expression depicting a negative or positive emotion is flashed briefly as a prime, and an emotionally neutral target (e.g., an ideograph) is then presented. Participants are asked to emotionally evaluate the target. Previous studies have reported that evaluations of the target were negatively biased by unconscious negative compared with positive primes (Murphy and Zajonc, 1993; Murphy et al., 1995; Rotteveel et al., 2001). This effect has been interpreted as evidence that unconscious emotion can be elicited and that it affects the evaluation of unrelated targets (Murphy and Zajonc, 1993).

However, the subliminal priming paradigm did not always yield robust and strong effects. Several previous studies reported either the null effects of subliminally presented facial expressions (e.g., Kemps et al., 1996; Raccuglia and Phaf, 1997; Paul et al., 2012) or even a contrast effect (Kobylinska and Karwowska, 2007). Although Murphy and Zajonc (1993) reported that affective priming is most potent when it is subliminal (also see, Sweeny et al., 2009), the use of a short presentation time for stimuli, which is necessary to prevent conscious awareness, may prevent the use of unconscious processing in response these stimuli. Furthermore, the optimal temporal threshold for the presentation of stimuli is difficult to assess under a subliminal condition (Pessoa, 2005). Therefore, it would be helpful to develop a new experimental method that would facilitate a stronger and more robust subliminal effect of emotional facial expressions to clarify the psychological mechanisms underlying this phenomenon.

The presentation of dynamic facial expressions may be relevant in this regard, as these are more natural and powerful cues in real-life social interactions than are static expressions. From an evolutionary perspective (Darwin, 1872), human minds are programmed to process dynamic facial expressions of conspecifics more efficiently than they process static ones, which are artificial signals or products of technology. Consistent with this notion, several behavioral studies have indicated that dynamic facial expressions induced more evident behavioral reactions, such as emotion elicitation (Sato and Yoshikawa, 2007a), perception (Yoshikawa and Sato, 2008), and facial mimicry (Sato and Yoshikawa, 2007b), compared with static ones. The advantages of using dynamic compared with static facial expressions to induce behavioral reactions have even been shown in newborn infants (Vinter, 1986). These data suggest the advantage of using dynamic versus static facial expressions to elicit behavioral reactions, including unconscious emotions.

Neuroscientific evidence has also suggested the appropriateness of dynamic presentations for eliciting unconscious processing of emotional facial expressions. Previous neuroimaging studies have shown that subliminal presentations of emotional facial expressions activated the amygdala (e.g., Whalen et al., 1998; Suslow et al., 2013), which is more active when viewing dynamic than static facial expressions (e.g., LaBar et al., 2003; Sato et al., 2004). Neuroimaging studies have also suggested that the processing of unseen emotional facial expressions is related to the subcortical visual pathway to the amygdala, including the superior colliculus (e.g., Morris et al., 1999, 2001), and the superior colliculus was shown to be more sensitive to dynamic compared with static visual information (Schneider and Kastner, 2005). Based on these findings, we hypothesized that dynamic presentations of facial expressions would enhance subliminal emotional effects.

**FIGURE 1:**
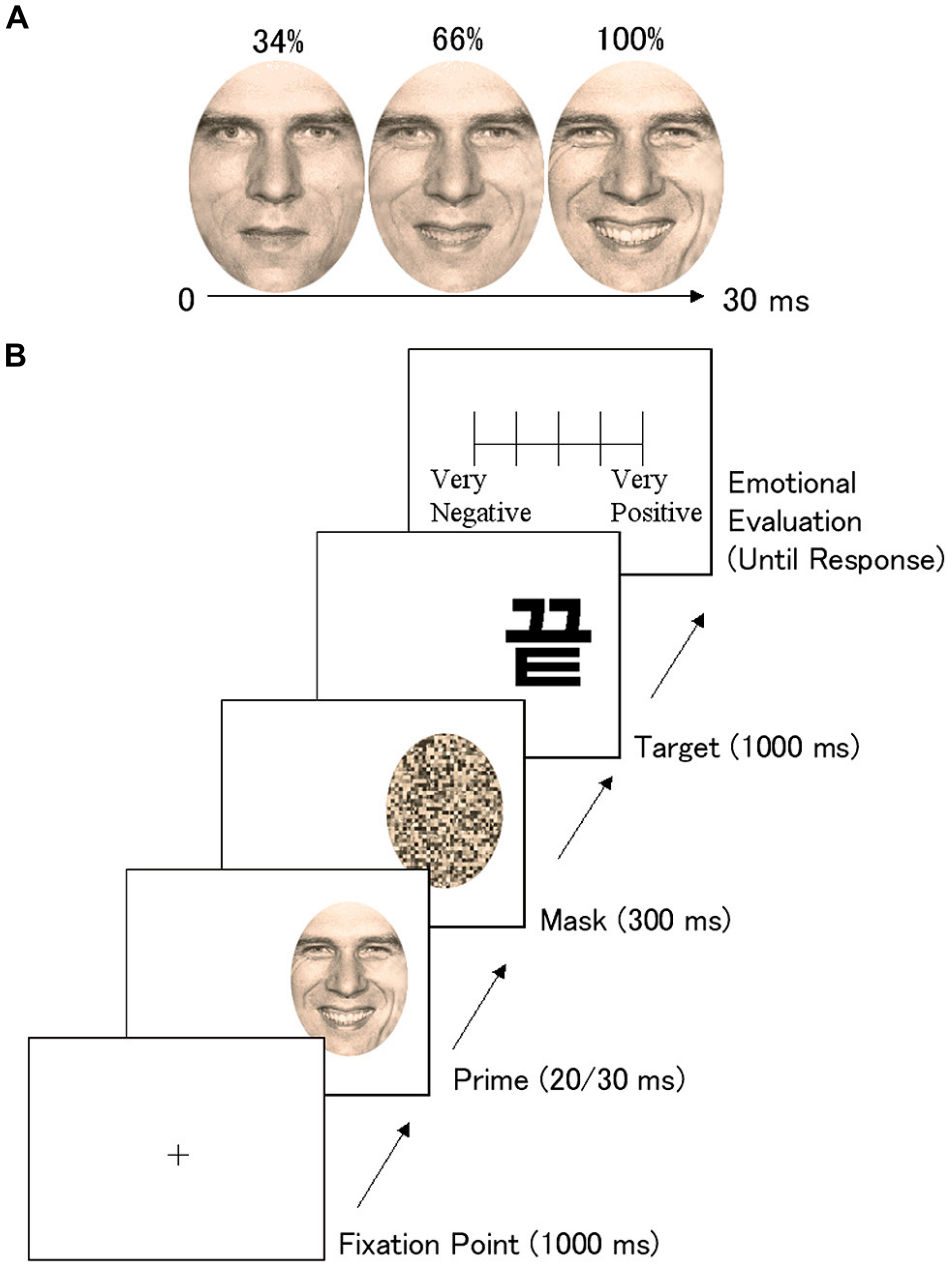
**An example of dynamic facial expressions **(A)** and the trial sequence **(B)****.

To test this hypothesis, we developed the dynamic subliminal affective priming paradigm. Fearful or happy facial expressions were presented dynamically or statically for 20 and 30 ms, followed by a mosaic mask (**Figure 1**). Then, nonsense target ideographs were presented, and participants evaluated the targets. Given that ample evidence indicates that the subcortical visual pathway is dominant in processing the peripheral compared with the central visual field (Waleszczyk et al., 2004), we presented the prime stimuli in the left or right peripheral visual field. To ensure the robustness of the phenomenon, we presented the stimuli at two different speeds. We predicted that fearful and happy facial expressions would affect participants’ reactions to the targets to a greater extent when they were dynamic compared with when they were static.

## MATERIALS AND METHODS

### PARTICIPANTS

Twenty-three healthy volunteers participated in Experiment 1 (10 females and 13 males, mean ± SD age, 21.6 ± 2.8 years), and 22 others participated in Experiment 2 (eight females and 14 males, mean ± SD age, 21.6 ± 2.4 years). All participants were Japanese, and none was familiar with Korean characters (the target ideographs). All participants were right handed, as assessed by the Edinburgh Handedness Inventory (Oldfield, 1971), and had normal or corrected-to-normal visual acuity. All were ignorant of the purpose of the experiment. Written informed consent was obtained from all participants after the experimental procedures had been fully explained. This study was approved by the local ethics committee of the Primate Research Institute, Kyoto University.

### EXPERIMENTAL DESIGN

The experiment was constructed as a within-subjects three-factorial design, with presentation condition (dynamic/static), emotion (negative/positive), and visual field (left/right) as factors.

### STIMULI

The raw materials of the prime stimuli were grayscale photographs of the faces of 10 Caucasian individuals (five females and five males) chosen from a standard set (Ekman and Friesen, 1976) depicting fearful, happy, and neutral expressions. Neutral expressions were used as the starting point to create morphing animations and to create a mosaic image. None of these faces was familiar to any of the participants. For the purpose of minimizing extraneous clues (e.g., hair), the faces were oval. The visual angles of the prime facial expression stimuli were 7.0^∘^ vertical × 5.0^∘^ horizontal.

Dynamic facial expressions were created from the photos using a morphing technique (**Figure 1**). First, based on neutral (0%) and emotional (100%) expressions, facial expressions with intensities of 34 and 66% were created using morphing software (FUTON System, ATR) implemented on a computer operating with Linux. This software has been used in several other studies (e.g., Sato et al., 2004; Sato and Yoshikawa, 2007a). Next, to create a dynamic video clip, 34, 66, and 100% facial expressions were presented in succession. In Experiment 1 (2), each image was presented for 6.7 (10) ms, and thus each clip lasted for 20 (30) ms.

Photographs of emotional facial expressions (100%) were presented as static expressions for 20 and 30 ms in Experiments 1 and 2, respectively. A mosaic image was created from a neutral facial expression by dividing the photos into a 50 × 40 grid and randomly reordering the pieces, rendering the resulting photograph unrecognizable as a face.

The target neutral ideographic stimuli were 40 Korean characters. These stimuli were shown to be emotionally neutral and used in a subliminal affective priming experiment in a previous study (Sato and Aoki, 2006). The visual angle of the target stimuli was 5.0^∘^ vertical × 5.0^∘^ horizontal.

### APPARATUS

Stimulus presentation was controlled by Presentation 14.9 (Neurobehavioral Systems) implemented on a Windows computer (HP Z200 SFF, Hewlett-Packard Company). The stimuli were presented on a 19-inch CRT monitor (HM903D-A, Iiyama) with refresh rates of 150 Hz (Experiment 1) and 100 Hz (Experiment 2) and a resolution of 1024 × 768 pixels. The refresh rate was confirmed by a high-speed camera (EXILIM FH100, Casio) with a temporal resolution of 1000 frames/s.

### PROCEDURE

The experiments were conducted individually. Participants were seated 0.57 m from the monitor. An emotional evaluation session preceded a forced-choice recognition session. After the forced-choice recognition session, participants were debriefed and asked whether they had consciously perceived the primes in the emotional evaluation session.

#### Emotional evaluation

A total of 80 trials (10 trials with 10 individuals’ faces under each of eight experimental conditions: 2 presentation conditions × 2 emotions × 2 visual fields) involving preference judgments were performed. The targets were randomly assigned to the experimental conditions and presented twice during the experiment. All trials were conducted in a pseudorandomized order. Participants initially participated in a block of 10 practice trials to become familiar with the procedure.

In each trial (**Figure 1**), a cross was initially presented for 1000 ms as a fixation point at the center of the visual field. A prime stimulus was then presented for 20 ms (Experiment 1) or 30 ms (Experiment 2) in either the left or the right visual field (the inside edge was 9.5^∘^ peripheral to the center); this was immediately followed by the presentation of a mask stimulus in the same place for 300 ms. The exposure duration of the prime and mask stimuli and the stimulus onset asynchrony (SOA) between them were determined based on data from previous subliminal studies (Esteves and Ohman, 1993) and the results of our preliminary studies. Then, the target ideograph was immediately presented at the same location for 1000 ms. Finally, the rating display was presented until the participant finished responding.

The participants were asked to gaze throughout the experiment at the location at which the fixation point had been presented. The participants’ task was to rate their preferences for the target ideographs using a five-point scale from “not at all” to “very much.” They were asked to respond by pressing keys with their right index finger.

#### Forced-choice recognition

As an objective approach to measuring the subliminal effect of the prime stimuli, the forced-choice recognition session was held after the preference sessions, as in previous studies (Murphy and Zajonc, 1993; Sato and Aoki, 2006). We randomly selected half of the stimuli under each experimental condition to reduce the number of trials, and a total of 40 trials (five trials under each of eight experimental conditions) were conducted.

In each trial, the sequence of events was presented in the same manner as in the preference session. Then, the two photos of emotional facial expressions, one of which had been presented as the prime in the previous trial, were presented in the upper and lower visual fields. The emotion of the two facial stimuli was identical (i.e., fear or happiness), and the participants judged which face had been presented before. This task was based on the assumption that participants who had acquired a visual awareness of faces would be able to select these faces based on low-level visual information. We did not use the facial expressions depicting different emotions because a previous study showed that a patient with blindsight had discriminated among facial expressions depicting different emotions in the absence of a conscious awareness of the faces (de Gelder et al., 1999), suggesting that people can discriminate among faces displaying different emotions based on unconsciously processed emotional information.

### DATA ANALYSIS

Data were analyzed using SPSS 16.0J software (SPSS Japan). The preference rating data were analyzed with three-way repeated-measures analyses of variance (ANOVAs) with presentation condition, emotion, and visual field as within-participant factors. Simple-effect analyses were performed as follow-up analyses (Kirk, 1994).

Our analysis of the forced-choice recognition data used one-sample *t*-tests to calculate the percentage of correct recognition responses. We then conducted paired *t*-tests comparing dynamic with static presentation conditions.

## RESULTS

### PREFERENCE EVALUATION

**FIGURE 2:**
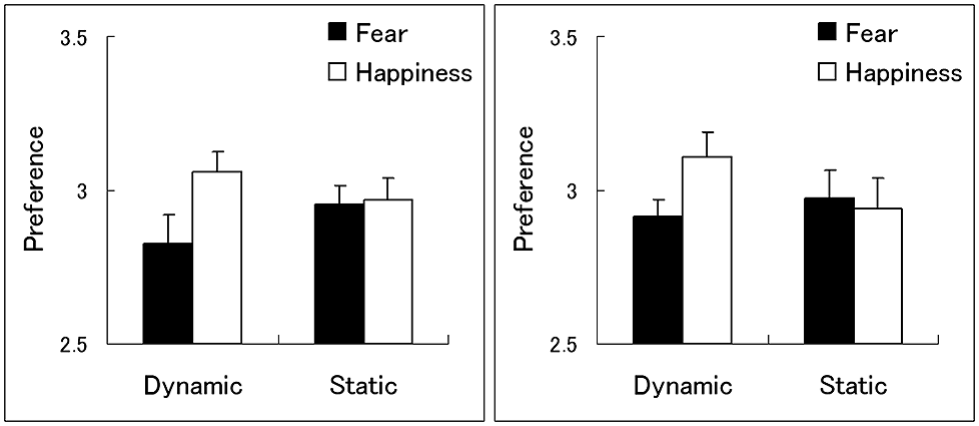
**Mean ratings (with SE) for emotional evaluation in Experiments 1 (left) and 2 (right)**.

In terms of the preference ratings in Experiment 1 (**Table 1**; **Figure 2**), the ANOVA with presentation condition, emotion, and visual field as factors revealed a significant interaction between presentation condition and emotion [*F*(1,22) = 5.54, *p* < 0.05], indicating that the subliminal emotional effects differed across presentation conditions. Additionally, the main effect of emotion and the interaction between presentation condition and visual field were marginally significant [*F*(1,22) = 3.47, *p* < 0.1; *F*(1,22) = 3.21, *p* < 0.1]. We found no other significant main effects or interactions (*p* > 0.1).

**Table 1:** Mean ratings (with SE) for emotional evaluation in Experiment 1 (upper) and 2 (lower).

Experiment	Dynamic				Static			
	LVF		RVF		LVF		RVF	
	Fear	Happiness	Fear	Happiness	Fear	Happiness	Fear	Happiness
1	2.87 (0.09)	3.07 (0.09)	2.80 (0.11)	3.04 (0.09)	2.88 (0.08)	2.87 (0.07)	3.07 (0.08)	3.06 (0.10)
2	3.03 (0.08)	3.17 (0.09)	2.80 (0.10)	3.05 (0.10)	2.91 (0.12)	2.92 (0.12)	3.04 (0.10)	2.96 (0.13)

*LVF, left visual field; RVF, right visual field.*

To clarify the subliminal emotional effect under each presentation condition, the simple main effects of emotion were analyzed to examine the interaction between presentation condition and emotion. The results revealed a significant simple main effect of emotion under the dynamic presentation condition [*F*(1,44) = 8.67, *p* < 0.01], indicating that subliminal dynamic presentations of fearful, compared with happy, facial expressions were associated with lower preference ratings for subsequent targets. This effect was not significant under the static presentation condition [*F*(1,44) = 0.01, *p* > 0.1].

Similar patterns were observed for the preference ratings in Experiment 2 (**Table 1**; **Figure 2**). The three-way ANOVA revealed that the interaction between presentation condition and emotion was significant [*F*(1,21) = 4.71, *p* < 0.05], indicating different subliminal emotional effects across presentation conditions. Additionally, the interaction between presentation condition and visual field was marginally significant [*F*(1,21) = 3.54, *p* < 0.1]. We found no other significant main effects or interactions (*p* > 0.1).

Follow-up simple-effect analyses were performed for the presentation condition × emotion interaction to test the subliminal emotional effect under each presentation condition. The simple main effect of emotion was significant under the dynamic presentation condition [*F*(1,42) = 4.76, *p* < 0.01] but not under the static presentation condition [*F*(1,42) = 0.63, *p* > 0.1], indicating that subliminally presented dynamic, but not static, fearful versus happy facial expressions reduced the preference for targets.

### FORCED-CHOICE RECOGNITION

The mean (±*SE*) percentage of correct recognition responses was 50.1% (±2.7) in Experiment 1 and 49.3% (±2.5) in Experiment 2. One-sample *t*-tests were performed to identify differences that exceeded the level of chance, and the results showed that the percentage of correct recognitions did not significantly differ from chance in either experiment (*t* < 0.84, *p* > 0.1). These results serve as the objective criterion that the primes had been presented subliminally under the present experimental conditions (Eriksen, 1960). We also tested for differences between dynamic and static presentation conditions and found no significant differences (paired *t*-test, *t* < 1.53, *p* > 0.1). The debriefing interview further confirmed that none of the participants had subjectively perceived the primes.

## DISCUSSION

Our data showed that dynamic presentations of facial expressions induced more evident subliminal emotional effects than did static presentations. These results are consistent with several behavioral studies showing the facilitative effect of various types of behavioral responses (e.g., Sato and Yoshikawa, 2007a). However, these previous studies investigated only the effect of the supraliminal presentation of dynamic facial expressions. To our knowledge, this is the first evidence demonstrating the facilitative effect of dynamic facial expressions on unconscious emotional processing.

We found no clear subliminal effects of emotional facial expressions under the static condition. This result is consistent with several previous studies reporting the null effects of subliminally presented static facial expressions (e.g., Paul et al., 2012), although other studies reported a significant effect in this regard (e.g., Murphy and Zajonc, 1993). This discrepancy may be attributable to methodological differences. In this study, we strictly controlled the presentation of subliminal stimuli by using carefully selected masking stimuli, software, and hardware; the success of this approach was confirmed with subjective and objective measures. These presentation conditions may not have been sufficient to activate the neurological mechanism underlying the subliminal emotional effects of static stimuli. However, our results indicate that dynamic presentations of expressions were able to elicit subliminal effects even though an excessively rapid presentation style may have precluded observation of the subliminal effects of static stimuli.

Our data showing an enhanced effect of subliminal presentations of dynamic versus static facial expressions have theoretical and practical implications for future research on unconscious emotional processing. Theoretically, our data suggest that unconscious emotional processing has a stronger impact on daily social interactions involving dynamic facial expressions than researchers have previously assumed based on data obtained using static presentations. It has been shown that unconscious emotional processing can modify various types of decisions and behaviors (Ferguson and Bargh, 2004), and actual social interactions involving dynamic facial expressions probably generate marked unconscious biases that impact decisions and behaviors.

Practically, our data suggest that researchers should use dynamic presentations to elicit more pronounced effect of subliminally presented emotional facial expressions. This technique would be helpful for detecting subtle effects, such as differences between normal and clinical groups. For example, a previous study tested the effect of subliminal affective priming in individuals with autism spectrum disorders and typically developing controls using static facial expressions (Kamio et al., 2006). Although the researchers found group differences, the results for typically developing controls did not clearly show the predicted pattern of affective priming. We speculate that dynamic presentations may facilitate the induction of a more robust effect of subliminal facial expressions. It would also be interesting to apply this technique to realistic situations. Several previous studies tested the effect of subliminal presentations of emotional pictures in realistic situations and reported null findings (e.g., Underwood, 1994). Based on such data, some researchers proposed that subliminal methods are not effective in realistic situations (e.g., Moore, 1992). Our results suggest that the more powerful effect of dynamic, compared with static, subliminal presentations of facial expressions may have the potential to modify decisions and behaviors in realistic situations.

Our data also offer clues about the neural mechanisms underlying the processing of subliminal facial expressions, suggesting that the unconscious processing of emotional facial expressions is implemented by a neural substrate sensitive to dynamic visual information. This finding fits well with the model based on neuroimaging data showing that the unconscious processing of emotional facial expressions may be implemented by subcortical visual input into the amygdala (Morris et al., 1999). Consistent with this model, one neuroimaging study has shown that amygdala activation was related to the emotional impact of subliminally presented facial expressions (Suslow et al., 2013), and an intracranial electroencephalographic recording study revealed that the amygdala was more active about 100 ms following stimulus onset in response to emotional than to neutral facial expressions (Sato et al., 2011). Anatomical studies in animals (Day-Brown et al., 2010) and humans (Tamietto et al., 2012) have shown that the amygdala receives visual input via subcortical pathways involving the pulvinar and superior colliculus, bypassing the cortical visual areas. With respect to the effect of visual motion information on these neural mechanisms, a neuroimaging study in humans (Schneider and Kastner, 2005) and ample evidence in animals (for reviews, see Stein, 1988; Waleszczyk et al., 2004) suggested that the superior colliculus was more sensitive to dynamic compared with static visual information. Several neuroimaging studies have also found that the amygdala was more active in response to dynamic versus static facial expressions (e.g., LaBar et al., 2003). Together with these data, our findings indicate that one of the important behavioral functions of amygdala activation by the subcortical visual pathway involves unconscious emotional processing in response to dynamic facial expressions.

Several limitations of the present study should be acknowledged. First, because we presented different event sequences after the presentation of facial expressions and varying masks across preference evaluations and forced-choice recognition tasks, the conscious awareness of faces observed in the former task may not have been reflected in the performance of the latter task. It is possible that differences in subsequent displays served as different backward masking conditions and produced different levels of conscious awareness. Changes in subsequent events may have also altered cognitive strategies, which may then have produced different levels of conscious awareness. Attempts to replicate the subliminal emotional effects observed for dynamic facial expressions using different threshold confirmation tasks (e.g., using the same event sequences with preference ratings but asking participants to label the emotions depicted by facial expressions) are needed to resolve this problem.

Second, we presented the faces of the same individuals repeatedly (although the number of trials was equal across experimental conditions), which may have influenced the preferential ratings. Several previous studies have reported that repeated subliminal presentations of stimuli increased the preference for those stimuli (e.g., Kunst-Wilson and Zajonc, 1980). Such data suggest that the overall ratings may have been inflated by the procedure used in the present study. Future research that manipulates the number of repetitions may be useful for examining this possibility.

Finally, we tested the effect of only dynamic presentations of emotional facial expressions because ample evidence has indicated that facial expressions can be processed unconsciously. Interestingly, some recent behavioral studies have shown that eye gaze was also processed without conscious awareness (Sato et al., 2007; Al-Janabi and Finkbeiner, 2012). Neuroscientific research suggested that the amygdala is related to the processing of eyes (Okada et al., 2008; Sato et al., 2009), as in the case of facial expressions. Another line of research has suggested that processing of emotional scenes can also be accomplished unconsciously and is related to the amygdala (Kubota et al., 2000; Gläscher and Adolphs, 2003). These data suggest the possibility that dynamic presentations may facilitate unconscious processing of these stimuli, which would be an important matter for future research.

In summary, our results showed that dynamic presentations of emotional facial expressions induced more evident emotional biases in evaluations of subsequent targets than did static ones. These results indicate that dynamic presentation of emotional facial expressions enhances unconscious emotional processing.

## Conflict of Interest Statement

The authors declare that the research was conducted in the absence of any commercial or financial relationships that could be construed as a potential conflict of interest.

## References

[B1] Al-Janabi S. Finkbeiner M. (2012). Effective processing of masked eye gaze requires volitional control. *Exp. Brain Res.* 216 433–443 10.1007/s00221-011-2944-0 22101495

[B2] Darwin C. (1872). *The Expression of the Emotions in Man and Animals.* London: John Murray 10.1037/10001-000

[B3] Day-Brown J. D. Wei H. Chomsung R. D. Petry H. M. Bickford M. E. (2010). Pulvinar projections to the striatum and amygdala in the tree shrew. *Front. Neuroanat.* 4:143 10.3389/fnana.2010.00143 PMC2991220 21120139

[B4] de Gelder B. Vroomen J. Pourtois G. Weiskrantz L. (1999). Non-conscious recognition of affect in the absence of striate cortex. *Neuroreport* 10 3759–3763 10.1097/00001756-199912160-00007 10716205

[B5] Eastwood J. D. Smilek D. (2005). Functional consequences of perceiving facial expressions of emotion without awareness. *Conscious. Cogn.* 14 565–584 10.1016/j.concog.2005.01.001 16091271

[B6] Ekman P. Friesen W. V. (1976). *Pictures of Facial Affect.* Palo Alto, CA: Consulting Psychologists Press, Inc

[B7] Eriksen C. W. (1960). Discrimination and learning withtout awareness: a methodological survey and evaluation. *Psychol. Rev.* 67 279–300 10.1037/h0041622 13697142

[B8] Esteves F. Ohman A. (1993). Masking the face: recognition of emotional facial expressions as a function of the parameters of backward masking. *Scand. J. Psychol.* 34 1–18 10.1111/j.1467-9450.1993.tb01096.x 8322040

[B9] Ferguson M. J. Bargh J. A. (2004). How social perception can automatically influence behavior. *Trends Cogn. Sci.* 8 33–39 10.1016/j.tics.2003.11.004 14697401

[B10] Gläscher J. Adolphs R. (2003). Processing of the arousal of subliminal and supraliminal emotional stimuli by the human amygdala. *J. Neurosci.* 23 10274–10282 14614086 10.1523/JNEUROSCI.23-32-10274.2003 PMC6741000

[B11] Kamio Y. Wolf J. Fein D. (2006). Automatic processing of emotional faces in high-functioning pervasive developmental disorders: an affective priming study. *J. Autism Dev. Disord.* 36 155–167 10.1007/s10803-005-0056-z 16523242

[B12] Kemps E. B. F. Erauw K. Vandierendonck A. (1996). The affective primacy hypothesis: affective or cognitive processing of optimally and suboptimally presented primes? *Psychol.**Belg.* 36 209–219

[B13] Kirk R. E. (1994). *Experimental Design: Procedures for the Behavioral Sciences* 3rd Edn. Pacific Grove, CA: Brooks/Cole

[B14] Kobylinska D. Karwowska D. (2007). The influence of lateral implicit visual affective stimuli on the evaluation of neutral stimuli in humans. *Acta Neurobiol. Exp.* 67 93–102 10.55782/ane-2007-1636 17474325

[B15] Kubota Y. Sato W. Murai T. Toichi M. Ikeda A. Sengoku A. (2000). Emotional cognition without awareness after unilateral temporal lobectomy in humans. *J. Neurosci.* 20:RC97 10.1523/JNEUROSCI.20-19-j0002.2000 PMC6772779 11000197

[B16] Kunst-Wilson W. R. Zajonc R. B. (1980). Affective discrimination of stimuli that cannot be recognized. *Science* 207 557–558 10.1126/science.7352271 7352271

[B17] LaBar K. S. Crupain M. J. Voyvodic J. T. McCarthy G. (2003). Dynamic perception of facial affect and identity in the human brain. *Cereb. Cortex* 13 1023–1033 10.1093/cercor/13.10.1023 12967919

[B18] Moore T. E. (1992). Subliminal perception: facts and fallacies. *Skept. Inq.* 16 273–281

[B19] Morris J. S. de Gelder B. Weiskrantz L. Dolan R. J. (2001). Differential extrageniculostriate and amygdala responses to presentation of emotional faces in a cortically blind field. *Brain* 124 1241–1252 10.1093/brain/124.6.1241 11353739

[B20] Morris J. S. Ohman A. Dolan R. J. (1999). A subcortical pathway to the right amygdala mediating “unseen” fear. *Proc. Natl. Acad. Sci. U.S.A.* 96 1680–1685 10.1073/pnas.96.4.1680 9990084 PMC15559

[B21] Murphy S. T. Monahan J. L. Zajonc R. B. (1995). Additivity of nonconscious affect: combined effects of priming and exposure. *J. Pers. Soc. Psychol.* 69 589–602 10.1037/0022-3514.69.4.589 7473021

[B22] Murphy S. T. Zajonc R. B. (1993). Affect, cognition, and awareness: affective priming with optimal and suboptimal stimulus exposures. *J. Pers. Soc. Psychol.* 64 723–739 10.1037/0022-3514.64.5.723 8505704

[B23] Okada T. Sato W. Kubota Y. Usui K. Inoue Y. Murai T. (2008). Involvement of medial temporal structures in reflexive attentional shift by gaze. *Soc. Cogn. Affect Neurosci.* 3 80–88 10.1093/scan/nsm027 19015098 PMC2569822

[B24] Oldfield R. C. (1971). The assessment and analysis of handedness: the Edinburgh inventory. *Neuropsychologia* 9 97–113 10.1016/0028-3932(71)90067-4 5146491

[B25] Paul E. S. Pope S. A. Fennell J. G. Mendl M. T. (2012). Social anxiety modulates subliminal affective priming. *PLoS ONE* 7:e37011 10.1371/journal.pone.0037011 PMC3355168 22615873

[B26] Pessoa L. (2005). To what extent are emotional visual stimuli processed without attention and awareness? *Curr. Opin. Neurobiol.* 15 188–196 10.1016/j.conb.2005.03.002 15831401

[B27] Pribram K. H. Gill M. M. (1976). *Freud’s “Project” Re-assessed: Preface to Contemporary Cognitive Theory and Neuropsychology.* New York, NY: Basic Books

[B28] Raccuglia R. A. Phaf R. H. (1997). Asymmetric affective evaluation of words and faces. *Br. J. Psychol.* 88 93–116 10.1111/j.2044-8295.1997.tb02623.x 9061894

[B29] Rotteveel M. de Groot P. Geutskens A. Phaf R. H. (2001). Stronger suboptimal than optimal affective priming? *Emotion* 1 348–364 10.1037/1528-3542.1.4.348 12901397

[B30] Sato W. Aoki S. (2006). Right hemispheric dominance in processing of unconscious negative emotion. *Brain Cogn.* 62 261–266 10.1016/j.bandc.2006.06.006 16899333

[B31] Sato W. Kochiyama T. Uono S. Matsuda K. Usui K. Inoue Y. (2011). Rapid amygdala gamma oscillations in response to fearful facial expressions. *Neuropsychologia* 49 612–617 10.1016/j.neuropsychologia.2010.12.025 21182851

[B32] Sato W. Kochiyama T. Uono S. Yoshikawa S. (2009). Commonalities in the neural mechanisms underlying automatic attentional shifts by gaze, gestures, and symbols. *Neuroimage* 45 984–992 10.1016/j.neuroimage.2008.12.052 19167506

[B33] Sato W. Kochiyama T. Yoshikawa S. Naito E. Matsumura M. (2004). Enhanced neural activity in response to dynamic facial expressions of emotion: an fMRI study. *Cogn. Brain Res.* 20 81–91 10.1016/j.cogbrainres.2004.01.008 15130592

[B34] Sato W. Okada T. Toichi M. (2007). Attentional shift is triggered by gaze without awareness. *Exp. Brain Res.* 183 87–94 10.1007/s00221-007-1025-x 17624520

[B35] Sato W. Yoshikawa S. (2007a). Enhanced experience of emotional arousal in response to dynamic facial expressions. *J. Nonverbal Behav.* 31 119–135 10.1007/s10919-007-0025-7

[B36] Sato W. Yoshikawa S. (2007b). Spontaneous facial mimicry in response to dynamic facial expressions. *Cognition* 104 1–18 10.1016/j.cognition.2006.05.001 16780824

[B37] Schneider K. A. Kastner S. (2005). Visual responses of the human superior colliculus: a high-resolution functional magnetic resonance imaging study. *J. Neurophysiol.* 94 2491–2503 10.1152/jn.00288.2005 15944234

[B38] Stein B. E. (1988). Superior colliculus-mediated visual behaviors in cat and the concept of two corticotectal systems. *Prog. Brain Res.* 75 37–53 10.1016/S0079-6123(08)60464-1 3055061

[B39] Suslow T. Kugel H. Ohrmann P. Stuhrmann A. Grotegerd D. Redlich R. (2013). Neural correlates of affective priming effects based on masked facial emotion: an fMRI study. *Psychiatry Res.* 211 239–245 10.1016/j.pscychresns.2012.09.008 23131525

[B40] Sweeny T. D. Grabowecky M. Suzuki S. Paller K. A. (2009). Long-lasting effects of subliminal affective priming from facial expressions. *Conscious. Cogn.* 18 929–938 10.1016/j.concog.2009.07.011 19695907 PMC2784103

[B41] Tamietto M. Pullens P. de Gelder B. Weiskrantz L. Goebel R. (2012). Subcortical connections to human amygdala and changes following destruction of the visual cortex. *Curr. Biol.* 22 1449–1455 10.1016/j.cub.2012.06.006 22748315

[B42] Underwood G. (1994). Subliminal perception on TV. *Nature* 370 103 10.1038/370103a0 8022477

[B43] Vinter A. (1986). The role of movement in eliciting early imitations. *Child Dev.* 57 66–71 10.2307/1130638

[B44] Waleszczyk W. J. Wang C. Benedek G. Burke W. Dreher B. (2004). Motion sensitivity in cat’s superior colliculus: contribution of different visual processing channels to response properties of collicular neurons. *Acta Neurobiol. Exp.* 64 209–228 10.55782/ane-2004-1507 15366254

[B45] Whalen P. J. Rauch S. L. Etcoff N. L. McInerney S. C. Lee M. B. Jenike M. A. (1998). Masked presentations of emotional facial expressions modulate amygdala activity without explicit knowledge. *J. Neurosci.* 18 411–418 9412517 10.1523/JNEUROSCI.18-01-00411.1998 PMC6793390

[B46] Winkielman P. (2010). Bob Zajonc and the unconscious emotion. *Emotion Rev.* 2 353–362 10.1177/1754073910375480

[B47] Yoshikawa S. Sato W. (2008). Dynamic facial expressions of emotion induce representational momentum. *Cogn. Affect. Behav. Neurosci.* 8 25–31 10.3758/CABN.8.1.25 18405043

[B48] Zajonc R. B. (1980). Feeling and thinking: preferences need no inferences. *Am. Psychol.* 25 151–175 10.1037/0003-066X.35.2.151

